# On Model-Based Transfer Learning Method for the Detection of Inter-Turn Short Circuit Faults in PMSM

**DOI:** 10.3390/s23229145

**Published:** 2023-11-13

**Authors:** Mingsheng Wang, Qiang Song, Wuxuan Lai

**Affiliations:** National Engineering Laboratory for Electric Vehicles, Beijing Institute of Technology (BIT), Beijing 100081, China; wangmingsheng08@bit.edu.cn (M.W.); 3120195251@bit.edu.cn (W.L.)

**Keywords:** fault diagnosis, inter-turn short circuit (ITSC) fault, transfer learning, Bayesian optimization, permanent magnet synchronous motors (PMSMs), convolutional neural networks (CNNs)

## Abstract

The early detection of an inter-turn short circuit (ITSC) fault is extremely critical for permanent magnet synchronous motors (PMSMs) because it can lead to catastrophic consequences. In this study, a model-based transfer learning method is developed for ITSC fault detection. The contribution can be summarized as two points. First of all, a Bayesian-optimized residual dilated CNN model was proposed for the pre-training of the method. The dilated convolution is utilized to extend the receptive domain of the model, the residual architecture is employed to surmount the degradation problems, and the Bayesian optimization method is launched to address the hyperparameters tuning issues. Secondly, a transfer learning framework and strategy are presented to settle the new target domain datasets after the pre-training of the proposed model. Furthermore, motor fault experiments are carried out to validate the effectiveness of the proposed method. Comparison with seven other methods indicates the performance and advantage of the proposed method.

## 1. Introduction

Permanent magnet synchronous motors (PMSMs) are widely used in home appliances, wind turbines, industrial, and electric vehicles because of their high efficiency, high power density, and good torque control performance [1,2]. With the diversification of applications, the reliability of PMSM is gradually gaining attention. Motor failures can lead to unplanned shutdowns and even disastrous results, particularly in high safety-critical systems [3]. Therefore, the fault diagnosis of PMSMs is crucial for the safety of systems to avoid catastrophic consequences.

Stator winding’s inter-turn short circuit (ITSC) faults are one of the most common and difficult-to-identify faults in PMSMs [4]. Additionally, without timely and proper treatment, PMSM can suffer from more serious ITSC faults or even open-circuit faults [5]. ITSC faults are formed by the insulation failure of a stator winding, usually caused by mechanical stress, thermal stress, overcurrent, and aging [6]. When an ITSC fault occurs, the short circuit point will form an additional circuit connection parallel to the faulty winding and coupled to the other windings and rotor magnets through flux linkages [7]. An overcurrent is then generated in the faulty winding, resulting in a large amount of additional heat from ohmic losses, which can further intimidate adjacent wires and even melt them. Due to the above description, in some cases, even a minor ITSC fault can quickly extend to adjacent conductors, easily developing a minor fault into a serious one [8]. Hence, it is very important to detect and manage ITSC faults in their early stages.

Data-driven fault diagnosis is a typical fault diagnosis method that uses historical data to establish fault patterns without any prior explicit models or signal characteristics, which makes it ideal for fault diagnosis of complex systems [9]. With the rapid development of sensor technology, the collection of data has become more and more convenient. The data-driven fault diagnosis methods are receiving increasing attention.

The ability to learn intelligently from large amounts of historical data is a key feature of data-driven fault diagnosis methods. The traditional data-driven fault diagnosis methods usually have two steps, the first is manual feature extraction and selection, and the second is fault classification. In the first step, manual features are usually designed based on signal processing methods, which rely heavily on the prior knowledge and experience of human experts. In addition, the well-designed features are suitable for a specific diagnostic task, and when dealing with a different task the processes of manual feature extraction and selection need to be re-executed. Therefore, the first step is time-consuming and laborious. On the other hand, various traditional machine learning fault diagnosis methods such as artificial neural networks (ANN), fuzzy systems, and support vector machines (SVM) have been widely used in the fault classification step [10]. However, it is very challenging to perform high-precision fault diagnosis of complex devices using this extremely shallow structured method. Currently, the usage of deep learning has led to a new area in the field of machine learning, which can overcome the above-mentioned drawbacks. It can automatically learn high-level and hierarchical representation features of the huge raw data [11]. Deep learning methods have been broadly adopted in the domain of fault diagnoses, such as convolutional neural networks (CNN), deep belief networks (DBN), recurrent neural networks (RNN), and sparse autoencoders (SAE) [12]. As deep learning methods can reduce the impact of manual feature extraction processes, they have great potential in fault diagnosis.

However, there are still some challenges with using deep learning-based methods. Firstly, most of the current methods suppose that the feature distribution of the training data set is identical to that of the test data set, which is not realistic in practical usage [13,14]. Therefore, they are not appropriate for dealing with actual fault diagnostic tasks. For instance, when training data and testing data are acquired from the facility under different operating conditions, the performance of the diagnosis model, which is trained on a specific working condition, may not be so satisfactory [12]. Secondly, the training of deep learning models needs a large volume of data, and in practice, the amount of data available for model training is often limited, which tends to restrict the performance of the model [15]. Thirdly, due to the small volume of the samples in fault diagnosis, the depth of the deep learning models is usually no more than 5, which will limit the performance of their final predictions [16]. Fourthly, the hyperparameter tuning of the deep learning model is time-consuming, particularly for those unfamiliar with the process of parameter optimization [17]. 

Many studies have proposed the transfer learning (TL) based method to overcome the aforementioned problems [18]. Transfer learning can take full advantage of the knowledge learned from the existing tasks (source domain) to facilitate the model training of the new but similar tasks (target domain), and has gained more and more attention in recent years, such as image recognition, text classification, and biometrics [14,19,20,21,22]. Especially in the field of fault diagnosis, transfer learning has been widely used in the fault diagnosis of mechanical equipment and has achieved remarkable outcomes [23]. Kavianpour et al. introduced a novel semi-supervised transfer learning method for bearing fault diagnosis to solve the challenges caused by insufficient labeling data or changes in working conditions in practical applications [24]. He et al. used a fine-tuning transfer learning method to adapt a pre-trained model of a deep autoencoder network for fault diagnosis of a gearbox with only 80 labeled samples in the target task [25]. Yang et al. proposed a deep-targeted transfer learning method based on different conditional label distributions, and the results show that cross-domain data can be aligned by following a designable adaptation trajectory [26]. Zhang et al. transferred the parameters and modified the structure of a shallow ANN trained by sufficient source data to a similar task, in which only a limited number of labeled samples are available [20]. Rezaeianjouybari et al. proposed a novel multi-source domain adaptation transfer learning method for rotary machinery fault diagnosis, which can adapt the domains at both feature level and task level, and the results demonstrate the advantages over state-of-the-art methods [27]. 

It is clear from the abovementioned studies that the transfer learning method not only reduces the parameter tuning and training time of the deep learning model, but also allows the deep learning model to have a better performance in tasks with different data distributions, and even achieves good results in tasks with a limited number of samples. Inspired by this, a model-based transfer learning CNN method is proposed in this paper for the ITSC fault diagnosis of PMSM. The key contributions of the paper are summarized below:

(1) A well-designed deep transfer learning method, termed transfer residual dilated CNN, was proposed for the fault diagnosis of ITSC in a PMSM. In the proposed method, dilated CNN is employed to learn the transferable features from the raw three-phase current of the stator. In addition, the residual connection is used to guarantee that the proposed deep learning model can obtain sufficient depth;

(2) A novel freeze and tune transfer strategy based on a pre-trained deep learning model is used for the fault diagnosis of ITSC under different operating conditions of the pre-trained model;

(3) The Bayesian optimization method is used to do hyperparameters optimization of the proposed model, which means the entire tuning of hyperparameters is done automatically.

The remainder of this paper is arranged as follows. Section 2 briefly reviews the related works. The proposed method is presented in Section 3. Section 4 presents the experiments carried out to verify the proposed method. Section 5 concludes this paper and gives an outlook for future work.

## 2. Related Work

In this section, the related work is introduced. It mainly contains two aspects, the derivation of the ITSC fault indicator for PMSM, and the introduction of transfer learning.

### 2.1. ITSC Fault in PMSM

The diagnosis of ITSC fault is very critical as overcurrent and overheating can cause more severe issues. However, in prior research, no indicator is particularly suitable to direct the severity setting of an ITSC fault in its early stage. In this article, a fault indicator is derived for directing the early-stage severity setting of an ITSC fault test.

**Figure 1:**
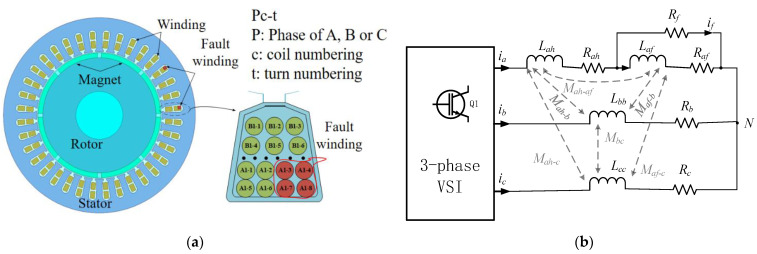
Schematic diagram of an 8-pole-36 slot PSMS with an ITSC fault in phase a. (**a**) Cross-section view. (**b**) Equivalent circuit model [29].

When an ITSC fault occurs in a PMSM, no matter where the shorting point is in a coil, the wires in the corresponding slot will be shorted accordingly, as the red wires are shown in Figure 1a [28,29]. Figure 1a presents a cross-sectional view of a PMSM with an 8-pole, 36-slot, and concentrated winding structure. The symbol Pc-t in the diagram indicates the unique number of each wire in a slot. Take A1-2 as an example; it indicates the second turn of wire in the first coil within phase A. When an ITSC fault occurs, an additional circuit is formed parallel to the same phase fault winding, as the equivalent circuit model shown in Figure 1b. From the model, it can be seen that the severity of the ITSC fault is influenced by the shorted turn ratio *μ* and the fault resistance *R_f_*. The equivalent circuit model can be expressed as

(1) $$\left[v_{abc,N}\right]=\left[R_{abc,f}\right]\left[i_{abc,f}\right]+\frac{d}{dt}(\left[L_{abc,f}\right]\left[i_{abc,f}\right]+\left[\lambda_{abc,f}\right])$$

$$\left[v_{abc,N}\right]={\left[\begin{array}{cccc} v_{a} & v_{b} & v_{c} & 0 \end{array}\right]}^{t}-{\left[\begin{array}{cccc} v_{N} & v_{N} & v_{N} & 0 \end{array}\right]}^{t}\;\left[i_{abc,f}\right]={\left[\begin{array}{cccc} i_{a} & i_{b} & i_{c} & i_{f} \end{array}\right]}^{t}\;\left[\lambda_{abc,f}\right]={\left[\begin{array}{cccc} \lambda \cos\theta_{e} & \lambda \cos(\theta_{e}-\frac{2}{3}\pi ) & \lambda \cos(\theta_{e}+\frac{2}{3}\pi ) & \mu \lambda \cos\theta_{e} \end{array}\right]}^{t}\;\left[R_{abc,f}\right]=\left[\begin{array}{cccc} R_{a} & & & \mu R_{a}\\ & R_{b} & & \\ & & R_{c} & \\ \mu R_{a} & & & \mu R_{a}+R_{f} \end{array}\right]\;\left[L_{abc,f}\right]=\left[\begin{array}{cccc} L_{aa} & M_{ab} & M_{ac} & \mu L_{aa}\\ M_{ab} & L_{bb} & M_{bc} & \mu M_{ab}\\ M_{ac} & M_{bc} & L_{cc} & \mu M_{ac}\\ \mu L_{aa} & \mu M_{ab} & \mu M_{ac} & \mu^{2}L_{aa}N_{c} \end{array}\right]$$

where *v_a_*, *v_b_*, and *v_c_* represent the terminal voltage of the access point phase A, phase B, and phase C, respectively. *v_N_* indicates the voltage at the neutral point. *i_a_*, *i_b_*, *i_c_*, and *i_f_* represent the phase current of phase A, phase B, phase C, and fault current in the shorted path. λ represents the amplitude of the permanent magnet flux linkage. *R_a_*, *R_b_*, *R_c_*, and *R_f_* represent the resistance of phase A, phase B, and phase C, and the fault resistance between the shorted turns. *L_aa_*, *L_bb_*, and *L_cc_* represent the self-inductance of phase A, phase B, and phase C. *M_ab_*, *M_bc_*, and *M_ca_* represent the mutual inductance between phase A, phase B, and phase C. *N_c_* represents the number of coils per phase, *N_t_* represents the number of turns in each coil, and *N_s_* represents the number of turns shorted in the fault phase. *μ* represents the shorted turn ratio, which can be expressed as *μ* = *N_s_*/*N_c_*·*N_t_*. 

According to Kirchhoff’s current law, the expression for the fault current can be derived from (1) as

(2) $$i_{f}=\frac{\mu (v_{a}-v_{N})+(\mu^{2}L_{aa}-\mu^{2}L_{aa}N_{c})\frac{di_{f}}{dt}}{\mu R_{a}+R_{f}-\mu^{2}R_{a}}$$

Since the amplitude of *v_N_* is much smaller than that of *v_a_* at the early stage of an ITSC fault, *v_a_* ≈ *v_a_* − *v_N_*. By defining *d*_1_ = *μRa* + *R_f_* − *μ^2^R_a_*, *d*_2_ = *μ^2^L_aa_* − *μ^2^L_aa_N_c_*, and *v_a_* = *V_a_*sin(*ωt*), the solution of (2) can be expressed as

(3) $$i_{f}(t)=e^{\frac{d_{1}t}{d_{2}}}\left(i_{f}(0)-\frac{\mu V_{a}\omega }{d_{2}\left(\omega^{2}+\frac{d_{1}{}^{2}}{d_{2}{}^{2}}\right)}\right)+\frac{\mu V_{a}\left(\omega \cos\left(\omega t\right)+\frac{d_{1}\sin\left(\omega t\right)}{d_{2}}\right)}{d_{2}\left(\omega^{2}+\frac{d_{1}{}^{2}}{d_{2}{}^{2}}\right)}$$

As *N_c_* > 1, *R_f_* ≥ 0, 0 ≤ *μ* ≤ 1, then *d*_1_ ≥ 0, *d*_2_ < 0, and in the early stage of an ITSC fault, |*d*_1_| >> |*d*_2_|, the ratio of *d*_1_ to *d*_2_ tends to infinity while the ratio of *d*_2_ to *d*_1_ tends to 0. Combining the above analysis and substituting the expressions of *d*_1_ and *d*_2_ into (3), the amplitude of *i_f_* can be expressed as

(4) $$I_{f}\approx \frac{\mu V_{a}}{\mu R_{a}+R_{f}-\mu^{2}R_{a}}$$

In addition, according to [30] the amplitude of the three-phase voltage is proportional to the rotor speed. Then the relationship among the rotor speed *ω_r_*, *R_f_*, *μ*, and *I_f_* can be described as

(5) $$I_{f}\propto \frac{\mu \omega_{r}}{\mu R_{a}+R_{f}-\mu^{2}R_{a}}$$

From (5), it can be seen that *I_f_* is directly influenced by *ω_r_*, *R_f_*, *μ*, and *R_a_*, where *R_a_* can be considered as a known parameter. Among the remaining parameters of the equation, only *ω_r_* does not impact the severity of the ITSC fault. If (5) is divided by *ω_r_*, an equation that relates only to the fault resistance and shorted turn ratio is obtained. which is expressed as

(6) $$FI=\frac{I_{f}}{\omega_{r}}\propto \frac{\mu }{\mu R_{a}+R_{f}-\mu^{2}R_{a}}$$

where *FI* denotes the fault indicator that can reflect the severity of an ITSC fault to some degree. When the PMSM is in healthy condition, the indicator is 0. When the PMSM is in an ITSC fault condition, the indicator is related to the fault resistance and shorted turn ratio. In the early stage of an ITSC fault, the indicator is almost invariant to the rotor speed, it increases as *μ* increases or *R_f_* decreases. Each severity of an ITSC fault can be treated as a unique combination of *μ* and *R_f_*. However, this indicator does not apply to the direct estimation of an ITSC fault in the operation of a PMSM, because it is very hard to measure *μ* and *R_f_*, during the operation of the motor. This does not mean that it is useless, as *μ* and *R_f_* are known parameters in fault setting, so this indicator can be treated as a severity indicator for the setting of an ITSC fault.

### 2.2. Transfer Learning

As a new branch of machine learning, transfer learning differs from many other traditional machine learning methods in that they are built on the hypothesis that the training and the testing data come from the same distribution [14]. For the sake of a better description of transfer learning, two basic concepts are introduced, namely domain and task [13]. 

Firstly, the domain D contains two critical elements, the marginal distribution *P*(*X*) and the feature space ***χ***, where *X* = {*x*_1_,…,*x*_n_} **∈ *χ*** denotes that *X* is a collection that contains samples from the features space ***χ***, for instance, the current signals that are collected from the motor in different operating and health conditions. Then, the two key components of a task usually include an objective function *f*(‧) and a labeled space *Y*, which corresponds to the classification method and health conditions of signals. In general, the objective function is not directly observable. Nevertheless, it can be studied from the pairs {*x*_i_, *y*_i_} of the training dataset. If the source domain data is noted as *D_S_* = {(*x_S_*_1_, *y_S_*_1_),…, (*x_Sn_*, *y_Sn_*)}, and the target domain data is noted as *D_T_* = {(*x_T_*_1_, *y_T_*_1_),…, (*x_Tn_*, *y_Tn_*)}. Then, the purpose of the transfer learning can be described as:

Considering the source domain *D_S_* and its learning task *T_S_*, a target domain *D_T_* and its learning task *T_T_*, the purpose of transfer learning is intended to help enhance the performance of the prediction function *f*(‧) on *D_T_* and *T_T_* by taking advantage of the knowledge learned in *D_S_* and *D_T_*, in which *D_S_* ≠ *D_T_*, *T_S_* ≠ *T_T_*.

In the field of fault diagnosis, it is a fact that the number of labeled samples in the training dataset is rather small compared with those that are used to train the ImageNet, ResNet-50, VGG-16, VGG-19, etc. [31]. Besides, the source domain usually is different from those of the target domain, while tasks of the source and target domains may be the same or different, namely *D_S_* ≠ *D_T_* or *T_S_* ≠ *T_T_*. This problem is very common in fault diagnosing when using the deep learning method to do the severity estimation of an ITSC fault [32]. A deep learning model is trained under one operating condition in the laboratory, and when using the trained deep model in practice, the operating may be different from that in the laboratory, and the targets both in the laboratory and practice may be the same or different. For this kind of problem, there are three alternative solutions to solve it, namely instance-based transfer, model-based transfer, and feature-based transfer [14]. Among them, model-based transfer learning methods that are very suitable for the above-mentioned scenario are based on the transport hypothesis that the tasks between the source domain and target domain have some knowledge in common at the model level [23]. This indicates that the transferable knowledge is well integrated into a pre-trained deep learning model whose parameters and architecture are generalized to help learn a robust target model.

## 3. Proposed Method

The proposed deep transfer learning method first preprocesses the collected raw three-phase current data. Then, a residual dilated CNN architecture is proposed to accomplish the fault diagnosis by employing the pre-processed data. By introducing transfer learning, the proposed architecture is capable of overcoming the challenges of realizing satisfactory diagnostic accuracy quickly in a different dataset with different operating conditions.

### 3.1. Data Pre-Processing

Usually, the data obtained from experiments are long-time 1-D waveform data, which contain many spurious electromagnetic interferences and high-frequency interference components that can have a serious impact on the accuracy of fault diagnosis. Therefore, signal pre-processing is needed for the collected raw data.

**Figure 2:**
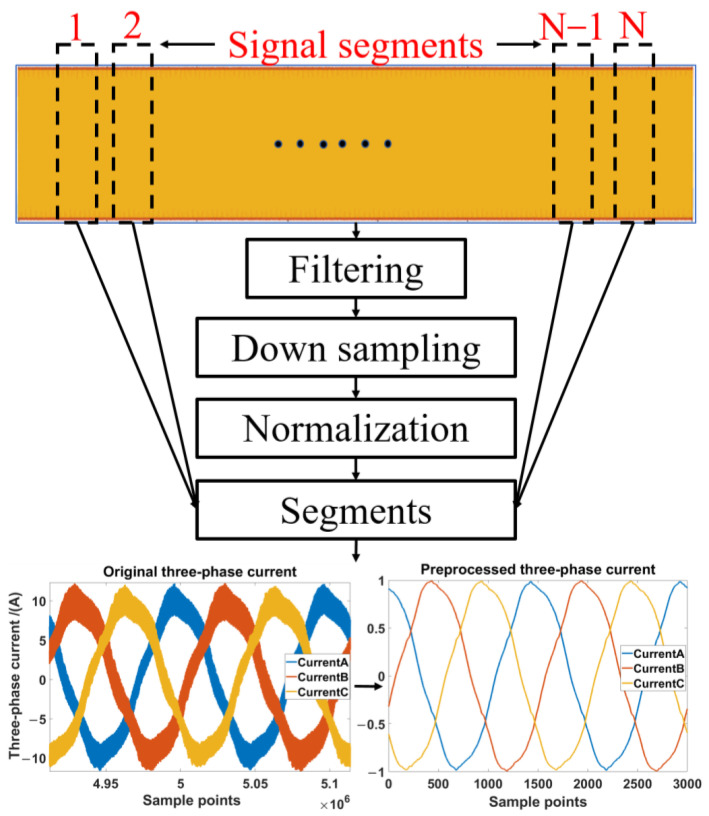
The procedure of the data pre-processing.

The procedure of the proposed data pre-processing is shown in Figure 2. First, the long 1-D raw signal is filtered by a 0-phase filter, which not only filters out unwanted interference components but also ensures that the phase of the filtered signal is the same as the original signal. Second, the filtered signal is downsampled to 15 kHz, which is the same as the switching frequency of the controller, in preparation for use in practice. Third, before feeding the signal to deep networks, the signal needs to be normalized so that the input data is restricted to a certain range (e.g., [0, 1] or [−1, 1]) for analysis. In this paper, we choose the mode of the maximum amplitude value as the denominator to normalize the signal, which is limited to the range of [−1, 1], as shown at the bottom of Figure 2. Fourth, there may be points at the beginning and end of the acquisition signal where the data is unstable due to experiments or acquisition equipment, so this part of the data needs to be removed and the whole data needs to be zero drift compensated. In the end, the three-phase current signals are cut into equal-length segments with the number of each segment being 1 × 3000 × 3.

To train and validate the proposed deep transfer learning network architecture, the signal segments need to be divided into two datasets based on the operating conditions, one for training and validating the proposed residual dilated CNN architecture, and the other for validating the proposed transfer learning method. The two datasets share the same fault labels but with different operating conditions.

### 3.2. Proposed Residual Dilated CNN Model

Typically transfer learning problems for images are based on mature network structures [33], such as the ImageNet, ResNet-50, VGG-16, VGG-19, etc. However, in this paper, the signal we adopted is 1-D current data, and the above-mentioned mature network is not applicable. Based on this, a residual dilated CNN model is proposed in this paper as a basis for transfer learning. The model is constructed in dilated convolution block, residual connection, and Bayesian optimization algorithm.

(1) Dilated convolution block

**Figure 3:**
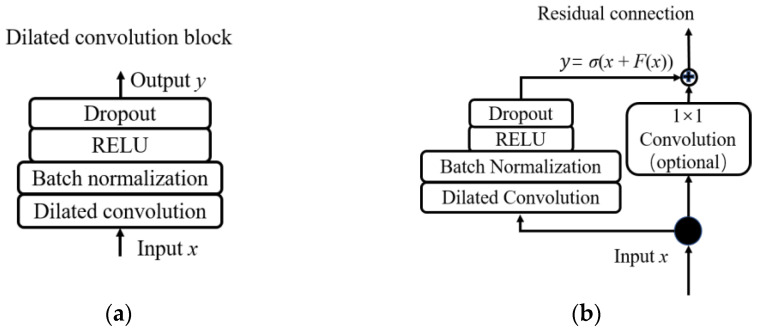
(**a**) A dilated convolution block. (**b**) Residual connection architecture.

The dilated convolution block consists of a dilated CNN, ReLU layer, batch normalization layer, and dropout layer, as shown in Figure 3a. The dilated CNN is a variant of the traditional CNN, which inherits the features of weight sharing and local connectivity and can optimize the result of the loss function by backpropagation algorithms [34]. The goal of the convolution operation is to extract hierarchical features from the input data at different levels. The deeper the convolution layer, the more complex the features obtained from the input data. Compared with conventional CNN, dilated CNN can eliminate the use of pooling layers, thus enlarging the receptive field without sacrificing the coverage or resolution, and enabling a fairly deep network structure possible [35]. For input data with 1-D signals ***S*** ∈***R****^n^* and denotes the kernel *f*: {0, 1, …, *k* − 1} → ***R***, then the dilated convolution *F* can be expressed as:

(7) $$F(x)=(S{*}_{d}f)(x)=\sum_{i=0}^{k-1}f(i)\cdot S_{x-d\cdot i}$$

where *x* stands for the input segments, *d* denotes the dilation factor, *k* stands for the filter size, and *x* − *d*‧*i* denotes the segment *x* traversing the elements of the *i*-th convolution operation. Hence, the dilated convolution means that the convolution operation is performed on the elements of the input data by kernels with filters separated by an interval of *d*. while *d* = 1, a dilated convolution becomes a conventional convolution. As the depth of the network increases, the dilated factor grows correspondingly, and the receptive field of the output layer becomes broader.

The rest layer of the dilated convolution block helps to enhance the performance of the network [29]. A normalization layer is employed to eliminate the possible gradient explosion or disappearance, and the method used here is Batch normalization. The activation layer is employed to expand the nonlinear representation capability of neurons, and the activation method we applied is a rectified linear unit (ReLU) to accelerate the process of training. To solve the overfitting difficulty of the network, a certain percentage of neurons and their connections can be randomly discarded, i.e., the dropout layer.

(2) Residual connection

The data used for the analysis of the ITSC fault of PMSM is three-phase current signals, which are extremely sensitive to electromagnetic interference and variable operating conditions. Besides, the fault features of an ITSC are extremely complicated making it difficult to extract sufficient features with shallow networks [36]. Thus, if we want to apply CNN to do the fault severity estimation of an ITSC, deeper architecture is needed, since usually the deeper the architecture of a CNN, the more complicated characteristics it can obtain. Whereas previous experiments have demonstrated that CNN suffers from degradation problems, i.e., the accuracy of the deep network converges to saturation or even degradation with the increase of the network depth. Namely, the increase in network depth decreases its performance, which is not caused by overfitting [37].

From the above analysis, we can conclude that it is not easy to train a CNN architecture well. Theoretically, the performance of the network should not degrade if the addition layer just repeats the features of the previous layer instead of learning new features, i.e., identity mapping. Inspired by this, the algorithm proposed in this paper uses a residual connection structure. For a residual connection, if the input of the architecture is denoted as *x*, the acquired features are marked as *F*(*x*). Then, the output of the residual connection can be defined as

(8) y=σ(x+F(x))

where *y* denotes the output of the residual connection, and σ stands for the activation function of the residual connection.

If the result of a residual connection is larger than 0, the performance of the network can be further improved by adding the network depth. On the other hand, if the result of a residual connection is 0, the newly added layer does not affect the performance of the network, namely identity mapping. Therefore, a deeper network can be developed using the residual connection to avoid degradation problems.

The residual connection is achieved by shortening the input and output of several layers. In a standard residual network, the output of several shorted layers is directly added to its input without any transformation. However, for the situation of 1-D CNN, an additional 1 × 1 convolution is employed to solve the tensor inconsistency problem between the input and output of the shorted layers, as shown in Figure 3b. 

(3) Bayesian optimization for hyperparameter tuning

The performance of the proposed model is heavily reliant on an optimal array of hyperparameters. However, the different hyperparameters are interrelated and it is hard to tune a suitable set of hyperparameters without experience; when it is possible, it requires a large amount of time [17,38]. In addition, when comparing several different algorithms, an automatic tuning function of hyperparameters would avoid the introduction of subjective intent making the comparison more objective. Therefore, it is necessary to introduce the function of hyperparameter tuning.

Bayesian optimization performs the evaluation and estimation of a mission by iterating and evolving a global statistical model with no explicit objection function [39]. Because of its fast optimization efficiency, it is widely applied. It is composed of two parts: the Bayesian statistical model and the acquisition function [16]. Bayesian statistical models employ prior observations and information to evaluate the hypothesis of the posterior distribution for the function to be optimized. The acquisition function is adopted to locate the sampling points or areas where the best solution is most likely to appear. In this paper, the Gaussian process is employed as the Bayesian statistical model and the Excepted Improvement is applied as the acquisition function.

**Figure 4:**
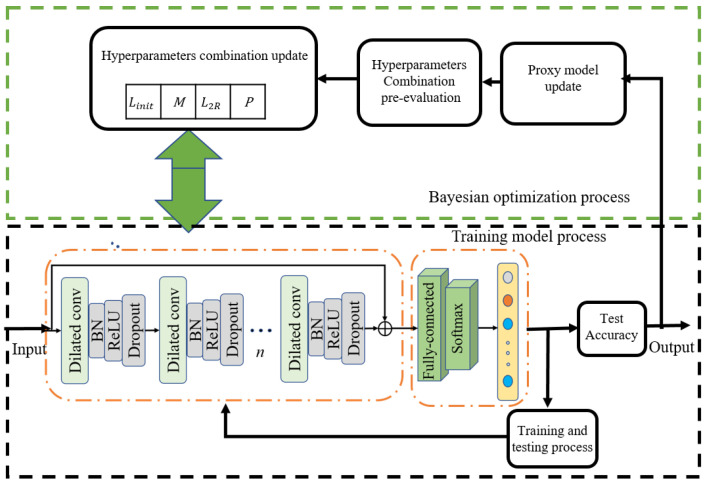
The process of hyperparameters tuning using Bayesian optimization.

The tuning process of hyperparameters with the Bayesian optimization algorithm is graphically demonstrated in Figure 4. The whole process consists of two parts: the training model process and the Bayesian optimization process [40]. The black box in Figure 4 is the model training process, which mainly achieves the training and testing of the proposed deep model. When the termination condition is reached, the training model process conveys the test accuracy of the model to the Bayesian optimization process. The green box in Figure 4 is the Bayesian optimization process, which mainly fulfills the initialization of the hyperparameters and then optimizes them according to the previous results. The hyperparameters to be optimized contain the InitialLearnRate (*L_init_*), Momentum (*M*), the L2Regularization (*L*_2*R*_), and the dropoutProb (*P*). During the implementation of the optimization, the two processes are iterated until the optimization termination condition is reached, and then the best result of the optimization is chosen as the output of the whole process.

(4) The architecture of the proposed model

**Figure 5:**
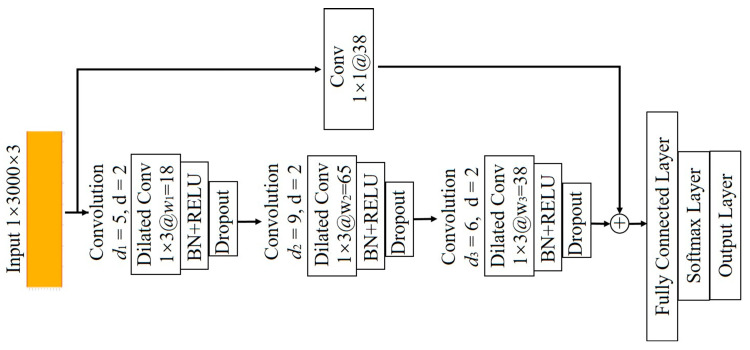
The schematic of the proposed model.

The schematic of the proposed model is shown in Figure 5. The model is composed of three main components: the input, the feature extraction, and the output. The input layer has three channels corresponding to the three-phase current signal, with each set of data segments having a length of 1 × 3000. The feature extraction part is constructed by stacking several dilated convolution blocks and combining them with the residual connection. The output, which is mainly responsible for classification and output, consists of a fully connected layer, a softmax layer, and an output layer. In this architecture feature extraction is the most important part, which is composed of three levels of dilated convolution blocks. The first level is responsible for extracting shallow features with a depth of 5 and a width of 18. The second level is in charge of extracting medium features with a depth of 9 and a width of 65. The third level takes the responsibility of extracting high features with a depth of 6 and a width of 38. All the dilated blocks share the same kernel size of 1 × 3 and the dilated factor of *d* = 2.

### 3.3. Proposed Deep Transfer Learning Architecture

**Figure 6:**
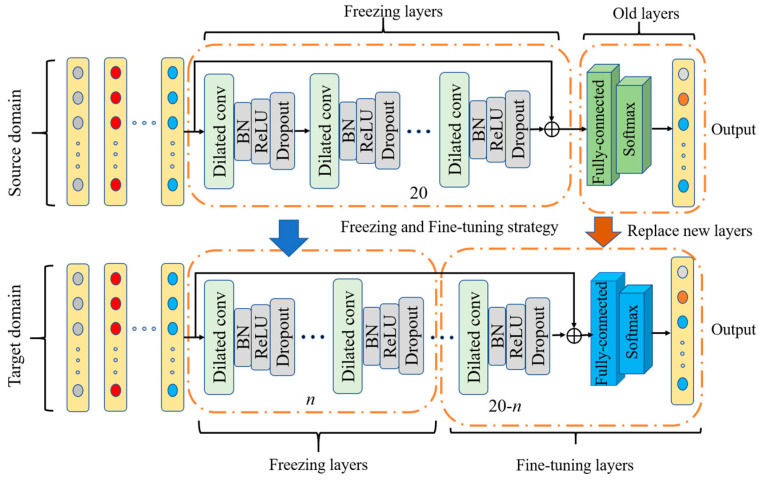
The framework of the proposed transfer learning method.

The framework of the proposed deep transfer learning model is shown in Figure 6. This transferable model is based on a fully pre-trained residual dilated CNN fault diagnosis model for the source domain dataset. The hyperparameters of the first *n* blocks in the pre-trained residual dilated model are frozen and transferred to the new construction of a model for samples of the target domain. The value of *n* ranges from 2 to 20 and the optimal value is obtained by the Bayesian optimization method. Then, (20 − *n*) new dilated convolution blocks, a new fully connected layer, a new softmax layer, and a new output layer are added to the new model to fit the class labels of the target domain. Compared with the former fault diagnosis networks, the depth of the proposed model is 24 blocks, which is rather deep. With deeper network layers and better feature extraction capability, the proposed deep transfer learning architecture would have a good performance on its final test accuracy and fault diagnosis [23,31,32]. In this architecture, the loss function for training the proposed model is the softmax cross-entropy, which is expressed as:

(9) $$H(y,p)=\sum_{i=1}^{N}-(y_{i}\log(p_{i}))$$

where *y* is equal to 1 when the current sample (sample *i*) falls into the designated class, if not it is 0, *p_i_* is the probability of the current sample (sample *i*) falling into the designated class, and *N* is the total number of the training samples. The complete procedure is described as follows:

First: Substitute the fault labels in the softmax layer based on the fault labels of the target domain.

Second: The parameters of the pre-sequence network layers are frozen. Then train the newly constructed network in a sample environment of the target domain.

Third: Tuning the subsequent connection layers using a small learning rate, which is designed for the generalization of the network transfer.

Fourth: Reducing the amount of fixed, frozen layers and moving them to the transfer connection layers. Likewise, using a small learning rate to tune the optimization of transfer connection layers.

Fifth: Replace the discriminative labels of the proposed model according to the samples of the test dataset, and then evaluate the performance of the model.

### 3.4. Performance Assessment with Cross-Validation

Cross-validation (CV) is a commonly used performance evaluation technique to obtain the reliability of fault diagnosis methods, among which K-fold CV is one of the most widely used CV methods [32]. In K-fold CV, the entire dataset is divided into K sub-datasets with a sample size of around equal cardinality N/K. Each sub-dataset in turn serves as a validating dataset, and the rest K-1 sub-datasets are applied for the training of the proposed fault diagnosis model.

In this paper, a tenfold CV is applied to realize the performance evaluation of the proposed deep transfer learning model on reliability. Let *P_v_* and $\hat{P_{v}}$ represent the actual labels and predicted labels of the validation dataset respectively, while *N_v_* represents the number of samples in the validation dataset. The validation accuracy of fault diagnosis is denoted as *Acc_v_*, while the validation accuracy of CV is denoted as *Acc_cv_*, and the expression can be defined as:

(10) $$\begin{array}{l} Acc_{v}=\frac{1}{N_{v}}(\sum_{i=1}^{N_{v}}1\{P_{v}=\hat{P_{v}}\})\\ Acc_{cv}=\frac{1}{10}(\sum_{i=1}^{10}Acc_{v}(i)) \end{array}$$

After completing the process of CV, the trained deep transfer learning model will be evaluated by another test dataset with a different operating condition. Similarly, let *P_t_* and ${\hat{P}}_{t}$ represent the actual and prediction labels of the testing dataset, while *N_t_* represents the number of samples in the testing dataset. Then, the final test accuracy (*Acc*) of the deep transfer learning model is defined as

(11) $$Acc=\frac{1}{N_{t}}(\sum_{i=1}^{N_{t}}1\{P_{t}=\hat{P_{t}}\})$$

### 3.5. The Procedure of The Proposed Method for Fault Diagnosis

**Figure 7:**
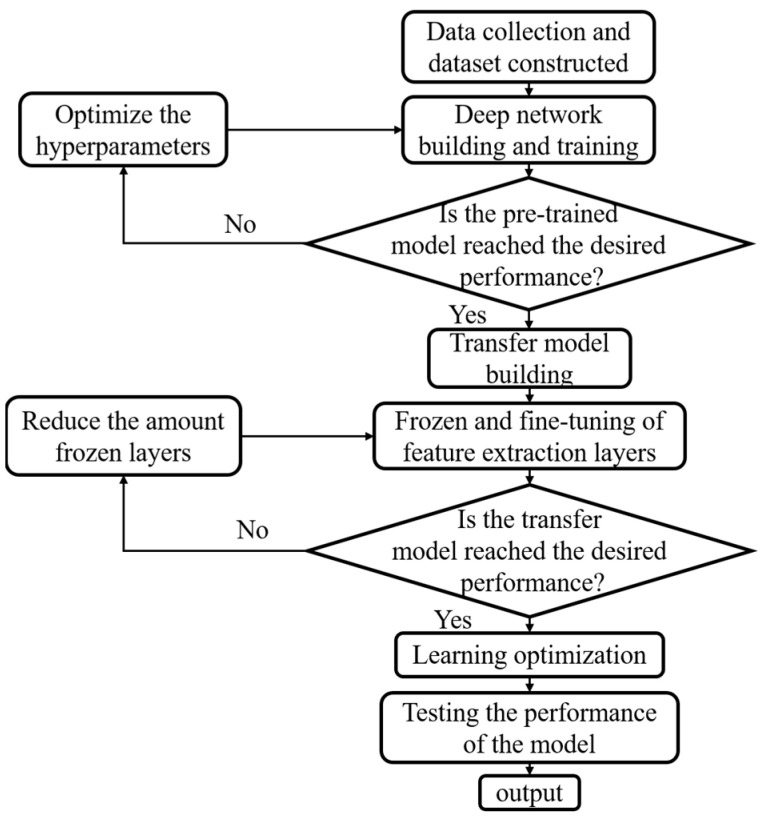
The flowchart of the deep transfer learning method.

The flowchart of the deep transfer learning method for the fault diagnosis of ITSC is shown in Figure 7. It mainly contains four processes in the whole framework.

(1) Data collection and dataset construction: The three-phase current signals of the tested PMSM are collected through current sensors and data acquisition equipment. Then, the collected data are divided into the source domain dataset and target domain dataset, in which the operating conditions of the two datasets are different.

(2) Build and pre-train the deep learning model: The residual dilated CNN model is built according to the given structure hyperparameters. Then, the proposed model is initialized randomly and pre-trained on the source domain dataset, and the training hyperparameters of the proposed model are optimized by Bayesian optimization.

(3) Build the deep transfer model: The deep transfer model is built by adopting some frozen layers of the pre-trained model, the newly fully connected layer, the softmax layer, and the output layer. 

(4) Fine-tune the deep transfer model: The hyperparameter tuning of the proposed deep transfer model is performed on the training dataset of the target domain, and the testing dataset of the target domain is employed to evaluate the performance of the deep transfer model on fault diagnosis. 

(5) Output the results: The performance of the proposed deep transfer model is fully evaluated by the method of ten-fold CV, and then, the best result and its corresponding hyperparameters are output as the final result of the proposed model.

## 4. Experiment and Results

### 4.1. Experiment Setup and Data Description

**Figure 8:**
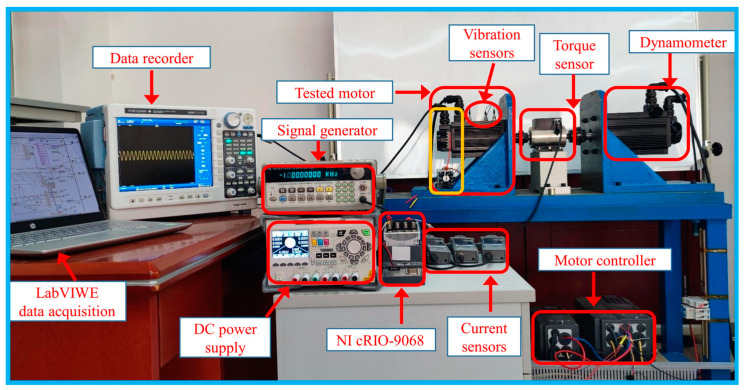
Experimental equipment diagram.

To verify the performance of the proposed deep transfer model in this article, experiments were conducted on PMSMs under a variety of operating conditions. The experiment setup was made up of a dynamometer, tested motors, current sensors, a data recorder, etc. as shown in Figure 8. The collected currents are captured by a DL850EA oscilloscope recorder with a sampling rate of 1MHz. The tested motors are driven by the controller at a switching frequency of 15 kHz. The tested motors are operated under the FOC control strategy at constant loads with speeds controlled by the dynamometer.

**Figure 9:**
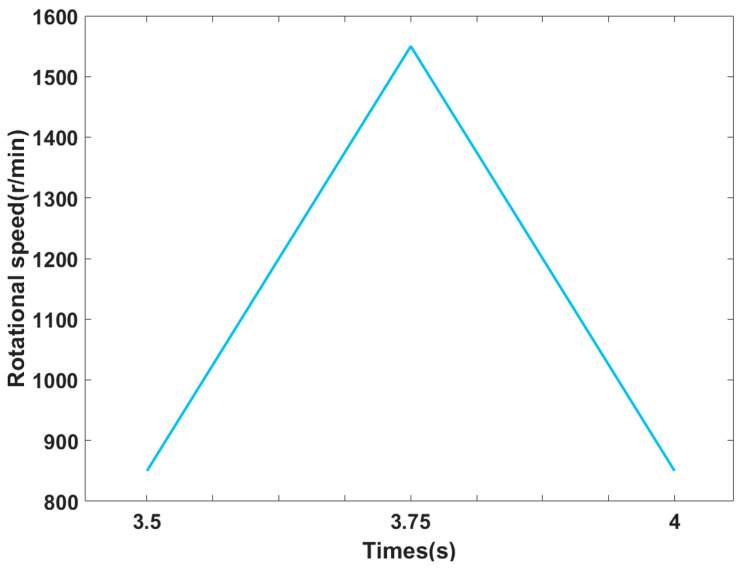
The schematic of the variable rotational speed [29].

The tested motors are operated under a variety of operating conditions, which are the combinations of two loads and five rotational speeds, as shown in Table 1. The two loads are both constant, while four of the rotational speeds are constant and one is variable. During the procedure of experiments, each rotational speed is carried out under every load. As shown in Figure 9, the variable rotational speed is set to a wide range to test the performance of the proposed deep transfer model under extreme operating conditions. 

**Figure 10:**
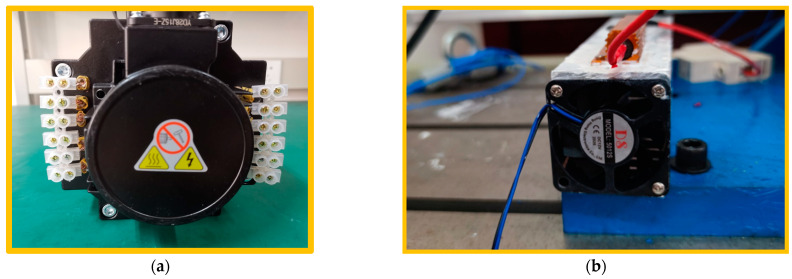
(**a**) The tested motor with shorted points on the winding. (**b**) The fault resistance and its heat sink.

The tested motor has four pairs of poles, 36 slots, concentrated winding, and wye-connection with 108 turns per phase. The major specifications of the tested motor are listed in Table 2. The ITSC faults were set in phase A of the tested motor. The lead wire terminals of the shorted points and the fault resistor with its heat sink are shown in Figure 10. Different shorted point corresponds to the different amount of shorted turns, while different fault resistances indicate the degree of insulation damage between the two shorted points.

As it is difficult to measure the shorted turn ratio and fault resistance during the running of a motor, the proposed fault index is not suitable to be used as a fault test indicator. However, in the procedure of the experiment, the fault index can be adopted to guide the severity setting for ITSC fault. In our experiments, we set up a total of 29 health states of a PMSM, 1 health state, and 28 ITSC fault states, as shown in Table 3 and Table 4. 

Every severity of the ITSC fault is a combination of fault resistance and shorted turn ratio. The fault is set on phase A winding by shorting two shorted points with a fault resistance. The data in Table 3 is used to verify the effect of the deep transfer model for different operating conditions under the same severity level and with a small sample number. The data in the source domain of Table 3 is utilized for training the proposed residual dilated CNN architecture, and the data in the target domain is utilized for testing the deep transfer model. As there are 10 operating conditions in the experiment, we adopted a ten-fold CV as the evaluation method to assess the performance of the proposed deep transfer model. When the training set is formed, 1 operating condition is selected for testing and the rest 9 are used for training. Make each operating condition traversed once in turn, keeping the total number of samples in source and target domains the same each time until the 10-fold CV is completed. The data in Table 4 is used to verify the effectiveness of the proposed deep transfer model for different severity levels under given operating conditions. The source domain will be the same as above mentioned in Table 3, the target domain is shown in Table 4. From Table 3 and Table 4, we can see that each label represents a fault severity of ITSC, which is named according to the shorted turn ratio and fault resistance. The label “HL” denotes the healthy state of the PMSM. There are 4 types of fault resistance, namely “R5”, “R1”, “R0.5”, and “R0.1”, which denote the fault resistances of 5 Ω, 1 Ω, 0.5 Ω, and 0.1 Ω respectively. In addition, “A1”, “A2”, “A3”, “A4”, “A5”, “A6”, and “A7” denote the shorted turn ratios of 2.78%, 4.6%, 7.41%, 8.3%, 10.2%, 13.8%, and 16.67% respectively. Moreover, the labels are listed in ascending order following the results calculated by (6).

**Figure 11:**
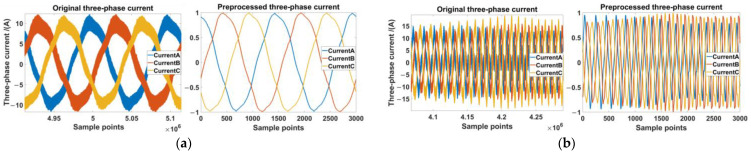
Comparison of the acquired three-phase currents before and after pre-processing, the left side of both figures shows the original signal and the right side shows the pre-processed signal. (**a**) The current that acquired in a healthy state at a constant operating condition of 150 rpm and 3.5 N·m. (**b**) The current that acquired in a faulty state of “A6R0.1” at a dynamic operating condition of the set speed and 3.5 N·m [29].

In this paper, the transfer learning method is used to solve two kinds of problems that will be encountered in the actual inter-turn short circuit fault diagnosis. One is the case that the operating conditions of the test data differ greatly from those of the training data and the number of samples is limited, and the other is the case that labels of the fault degree differ greatly from that of the training data but the amount of data is sufficient. Based on this premise we divided the collected dataset into three parts, one source domain data with two target domain data, as shown in Table 3 and Table 4. The comparisons between the signals before and after the pre-processing are shown in Figure 11. The total number of samples in Table 3 is 20,400, and 1200 for each label. Since there are 10 operating conditions under each label, the number of samples for each operating condition under this label is 120. Moreover, one of the problems to be solved is the fault diagnosis of ITSC under a different operating condition and with a small sample number. The data samples are divided into source and target domains, the source domain contains 18,360 samples in total, while the target domain contains 2040 samples. For each fault label in the source domain, there are 754 are for training and 326 are for testing. Similarly, for the target domain, there are 86 are for training and 34 are for testing. The samples in the target domain are collected under different operating conditions than the samples in the source domain. The samples in the source domain are used to train the proposed residual dilated CNN architecture, and the samples in the target domain are used to test the performance of the proposed deep transfer model. As for the fault diagnosis of ITSC for the target domain of different severity levels, the data configuration is shown in Table 4, there are 15,600 samples in total. Since there are 13 labels in Table 4 for the target domain of new different levels, the number of samples for each label is 1200, of which 840 samples are used for target domain training and 360 samples are used for testing. In addition, the data in Table 4 shares the same source domain as the data in Table 3.

### 4.2. Results and Comparison

The proposed network architecture is pre-trained after the construction of the dataset to obtain the deep learning model to be transferred. Throughout the pre-training process, the hyperparameters tuning process of the deep learning model is implemented by the Bayesian Optimization algorithm. The hyperparameters to be optimized are 4 training parameters, namely *L_init_*, *M*, *L_2R_*, and *N*. Where *L_init_* denotes the initial learning rate, *M* denotes the momentum of the model, *L_2R_* denotes the regularization method using the L2 norm, and *N* represents the blocks of the pre-trained model to be frozen. As can be seen from the previous section, the feature extraction layer of the pre-trained model adopts a three-stage structure, different values of *N* correspond to different feature extraction layers in the pre-trained model being frozen. When *N* takes 1 it means that only the first segment of the feature extraction layer is frozen. When *N* takes 2 it means that the first two segments of the feature extraction layer are frozen. When *N* takes 3 it means that all the feature extraction layers are frozen and only the full connectivity layer and the classification layer undergoes parameter update. When *N* takes 4 it means that none of the feature extraction layers are frozen and the parameters of all network layers need to be updated. The data types, search intervals, and best results of the optimized hyperparameters are shown in Table 5, where “Transform” denotes whether the corresponding hyperparameters will be optimized in a logarithmic scale or not. 

**Figure 12:**
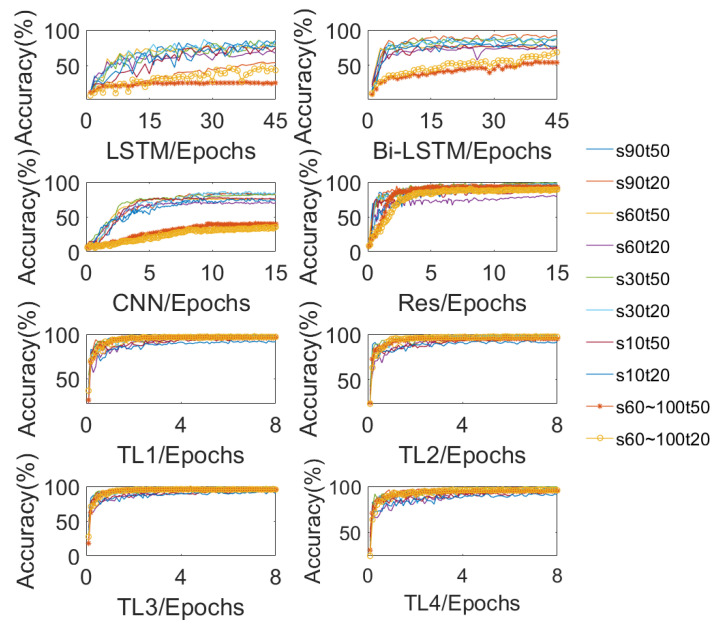
Comparison of the ten-fold CV evaluation results obtained by the 8 compared methods.

Transfer learning is performed after the completion of pre-training of the deep learning model. The dataset for transfer learning contains two types, a small sample dataset with the same fault severity (fault label) as the pre-training dataset but different operating conditions, and a large sample dataset with different severity levels (fault labels) from the pre-training dataset. A ten-fold CV evaluation method is adopted for the small sample cases with the same severity levels but different operating conditions as the pre-trained samples. Moreover, the experimental results are compared with four state-of-the-art deep learning algorithms currently for processing time series signals and the transfer learning methods for the case where n takes the remaining three values. The results are shown in Figure 12. The compared methods include two RNN methods, namely, LSTM and Bi-LSTM, two CNN methods, namely, conventional CNN and CNN with dilated convolution and residual architecture (Res), and four transfer learning methods which are applied based on the Res architecture. For different values of *N*, the transfer learning methods are denoted as TL1, TL2, TL3, and TL4, where TL1 is the proposed method. 

For the case of a small sample dataset with the same fault severity but different operating conditions, each method is validated for 10 different operating conditions, i.e., one of the operating conditions is selected as the testing dataset, and the rest of the operating conditions form the training dataset. To ensure the objectivity of the comparison, hyperparameters of the methods being compared are tuned using the Bayesian Optimization algorithm, the maximum number of optimizations is set to 80, and the result with the highest test accuracy is selected as the final output concerning each method. The figure shows the variation curves of the test accuracy during the training process with different operating conditions as the testing dataset, and each curve represents one operating condition respectively. Since RNN networks are difficult to train, they require more training epochs, which are set to 45, the training epochs of CNN and Res are set to 15, and the training epochs of four transfer learning methods are set to 8. From the figure, it can be seen that the transfer learning methods have advantages over the other compared methods in terms of both convergence speed and the final test accuracy. In terms of overall convergence speed, the CNN models outperform RNN models, and final test accuracy has mutual advantages and disadvantages. Besides, the recognition difficulty of dynamic operating conditions is higher than that of constant operating conditions. The average accuracy and training time for the ten-fold CV evaluation method of each algorithm are shown in Table 6. As can be seen from the table, these four transfer learning algorithms achieve far better results than the rest both in constant and dynamic operating conditions with fewer training epochs. Because the transfer learning methods can achieve better results in fewer training epochs, the training time for the four transfer learning algorithms is the least among all the methods. In terms of the average training time of the four transfer learning methods, the more layers that are frozen, the less time is used for training accordingly, which is in line with the setting. Among the four transfer learning methods, the TL1 method proposed in this paper has the highest average test accuracy and the smallest standard deviation, outperforming other methods compared. To summarize, in this application scenario, the proposed transfer learning method not only makes full use of the features learned by the pre-trained model, thus saving training time but also fine-tunes the parameters according to the new task, thus obtaining better performance in the target task.

**Figure 13:**
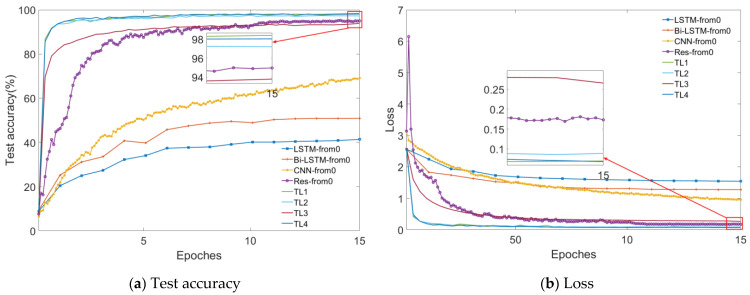
The trend of the test accuracy and loss with increasing training epochs for the compared methods.

For the case where there are a large number of data samples with different severity levels (fault labels) to the pre-training dataset, the proposed method is used and compared with the seven methods mentioned above. Due to the increased volume of data, the number of training epochs was set to 15. The trend of the test accuracy and loss with increasing training epochs for all methods in this paper is shown in Figure 13. The test accuracy and loss of each method on every epoch are saved during the whole training process. The hyperparameters of each method are optimized by Bayesian optimization and the best results are selected to do the comparison. 

Figure 13a is the trend of the test accuracy with increasing training epochs, and Figure 13b is the trend of the loss with increasing training epochs. The trends of these methods in the two figures are the same in terms of overall trends. It can be seen from Figure 13a that the four transfer learning methods have an unrivaled advantage over the rest of the compared methods in terms of the rising rate. The Res is secondary to the transfer learning methods on the rising rate. In terms of the final test accuracy, the four transfer learning methods and the Res are far more accurate than the other compared methods. When comparing the Res with the four transfer learning methods, it can be noticed that the final test accuracy of Res exceeds that of TL3, which suggests that not fine-tuning the parameters of the feature extraction layer in the presence of large amounts of data will limit the performance of the model in a new target task. Because of the increased number of training epochs, the performance of TL4 is very close to that of TL1 on final test accuracy and loss. The LSTM, Bi-LSTM, and CNN not only rise slowly but also give poor results in terms of final test accuracy. The three methods have the potential to improve the final accuracy but need more training epochs, which will be time-consuming. 

**Figure 14:**
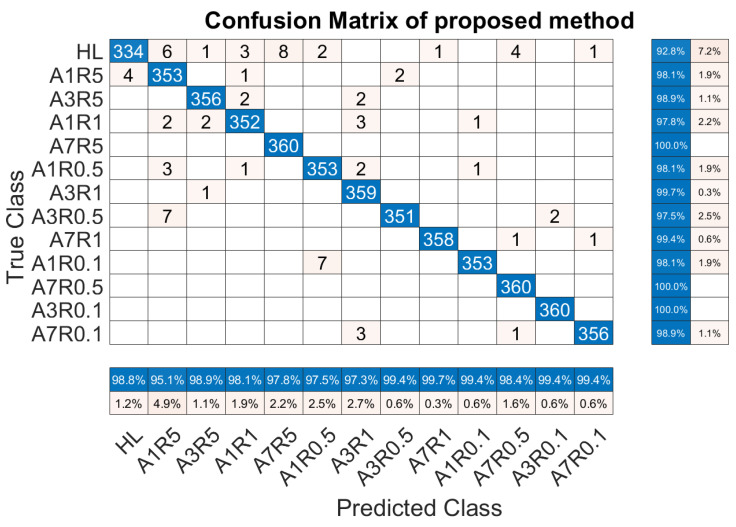
The confusion matrix of the proposed method.

The final test accuracy (ACC) of the transfer learning method is 98.40%, which is the best among the compared eight methods. To give a more detailed analysis, the confusion matrix of the final test accuracy is presented in Figure 14. “True Class” is the real label of the tested data, and “Predicted Class” is the label predicted by the transfer learning method. All labels are ordered in increasing sequence according to the severity calculated by (6). The numbers on the diagonal in the matrix indicate the number of samples on which the predicted labels and actual labels can correspond, i.e., the number of correct predictions for that label. The numbers outside the diagonal in the matrix indicate the number of samples that are incorrectly predicted. The label corresponding to the horizontal axis is the actual label type for that sample, and the label corresponding to the vertical axis is the type that is incorrectly predicted. Based on the above description, it can be seen that there are 4 cases in the confusion matrix. For one kind of label, the samples on the diagonal in the matrix are called true positives (TP), and the rest labels on the diagonal are true negatives (TN). Samples that do not belong to the current label but are predicted to be it are called false positives (FP), and samples that belong to the current label but are predicted to be others are called false negatives (FN). For each true label, the percentage of TP in a row is the precision ratio (*p*). For each predicted label, the percentage of TP in a column is the recall ratio (*r*). The precision ratio for each true label is at the rightmost of the matrix while the recall ratio for each predicted label is at the bottom of the matrix.

In a large amount of data, the precision ratio and recall ratio are regulated by each other. The *F*1 score takes into account the impact of both the recall ratio and precision ratio, which can better illustrate the capability of the method. Thus, to provide a more comprehensive assessment of the result, the *F*1 score is imported for evaluation. The adopted assessment metrics are expressed as

(12) $$\begin{array}{l} \mathrm{ACC}=\frac{TP+TN}{TP+TN+FP+FN}\\ r=\frac{TP}{TP+FN}\\ p=\frac{TP}{TP+FP}\\ F1=\frac{2p\times r}{p+r} \end{array}$$

For a comprehensive comparison of the best performing 5 out of 8 methods, the final test accuracy of each method, the *F*1 score of each method under every label, and the total training time of each method are listed in Table 7. From Table 7 we can note that the final test accuracies of the five methods are 94.94%, 98.40%, 97.22%, 93.76%, and 98.03% respectively. The proposed method not only has the best performance in the final test accuracy but also the *F*1 score corresponding to each label except “A1R5” and “A7R0.1”. In addition, in terms of time consumption, TL4 is close to Res, and the training time of the rest three transfer learning methods is related to the number of frozen layers. In terms of the average training time of the four transfer learning methods, the more layers that are frozen, the less time is used for training accordingly, which is in line with the setting. 

**Figure 15:**
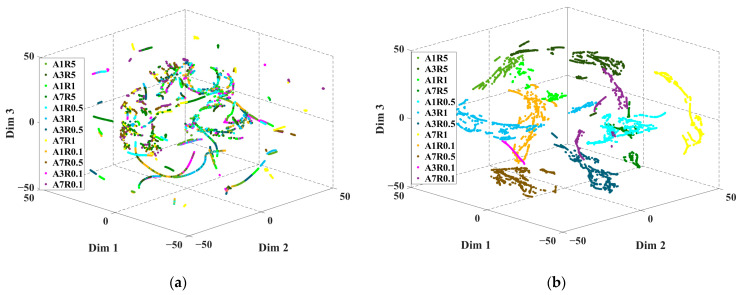
3-D visualizations of high-dimensional feature maps at different layers in the transfer learning method. The different colors in the feature map indicate different severity levels and each point represents an individual segment. (**a**) The feature map of the input layer. (**b**) The feature map of the last layer.

It can be observed that every method suffers from the same “false alarm” and “concealed alarm” problems when analyzing Figure 14 and Table 7. The former indicates the misclassification of health labels as fault labels, and the latter indicates the misclassification of fault labels as health labels, with the latter resulting in a catastrophic problem. This phenomenon is more significant when the severity is mild and less obvious when the severity is severe. This may be because when the fault severity is light, the difference between the fault characteristics is small, which increases the difficulty of distinguishing the severity of the fault. As the severity of the fault increases, the fault characteristics become more and more obvious, the difficulty of fault identification decreases, and the accuracy of fault detection increases. To validate this and visually represent the performance of transfer learning in fault feature learning, the features of the input layer and the last layer, which are displayed by the t-distribution stochastic neighbor embedding (TSNE) algorithm in a 3-D visual, are shown in Figure 15. 

To make the fault features pictorial and streamline the comparison, the fault feature dimensions of the original signal and the final layer are simplified to three dimensions, as presented in Figure 15. There are 13 different severity levels in the feature map, which are indicated by colors correspondingly. It can be seen in Figure 15a that the fault features of different severity levels are disorganized and heavily overlapped, which makes it extremely challenging to directly implement the classification of severity levels from the raw data. After the training of the proposed network, the distinction between fault features is very clear and easy to distinguish, as demonstrated in Figure 15b. It also can be learned from Figure 15b that the spacing between the fault features is small when the fault severity level is mild, and gradually becomes larger as the fault severity increases. This also verifies the phenomenon that the misclassification in the confusion matrix is more obvious when the fault severity is mild and the test accuracy gradually grows as the fault severity increases. As the adopted samples are time series signals, the transfer learning method can be applied for a successive diagnosis of the acquired signals in actual usage. With a test accuracy of 98.40% and an *F*1 score of over 95.7% for each label, the chance of two continuous misclassifications is lower than 0.1%. Therefore, the improvement of test accuracy along with the problem of “false alarm” and “concealed alarm” can be overcome by combining the diagnosis results of consecutive sample signals.

## 5. Conclusions

In this paper, a transfer learning method for ITSC fault diagnosis was proposed based on a Bayesian optimized residual dilated CNN model. The prior knowledge and proposed fine-tuning strategy enhanced the diagnostic performance of the pre-trained model for new target domain datasets. To begin with, the receptive domain of the model was extended utilizing dilated convolution. Then, residual architecture was employed to surmount the degradation problems in deep models. Afterward, the Bayesian Optimization method was launched to address the hyperparameter tuning issue of the proposed model. Moreover, after the construction of the proposed model, pre-training was executed on it. In the next part, a transfer learning framework and strategy were proposed to address new target domain datasets. Furthermore, motor fault experiments were carried out to get new datasets. Four transfer learning methods and the other 4 state-of-the-art deep learning methods for processing time series signals were applied to the new datasets. The results show that the proposed transfer learning method not only provided the best performance in the case of a small sample dataset with the same fault severity but different operating conditions but also in the case where there are a large number of data samples with different severity levels to the pre-training dataset. The results show that the proposed transfer learning method provides the best performance in both small sample datasets with different operating conditions and large sample datasets with new fault severity.

Although this paper has achieved certain achievements, there are still two issues that need to be further addressed. Firstly, even though the transfer learning method can save a lot of time, the method is still implemented offline and cannot achieve real-time diagnosis online. Second, the data used for training are all labeled data, which are difficult to obtain directly in practical applications. Therefore, the next research direction will be to study the adaptive training of the model for unlabeled data and to achieve real-time online fault diagnosis.

## Figures and Tables

**Table 1:** Operating conditions of the PMSM to be tested.

Case	1	2	3	4	5
Speed (rpm)	150	450	900	1350	850–1550–850
Torque (N·m)	3.0				
	7.5				

**Table 2:** Specifications of the PMSM.

Parameters	Values
Pole pairs	4
Power	2.3 kW
Rated torque	15 N·m
Rated current	9.5 A
Rated speed	1500 rpm
Line-line resistance	1.1 Ω
Line-line inductance	4.45 mH
Voltage constant	114 V/1000 r/min

**Table 3:** Fault data description 1.

Label	Fault Setting		Sample Size				
	Fault Resistance (Ω)	Shorted Ratio (%)	Source Domain		Target Domain		
			Train	Test	Train	Test	Total
HL	Inf	0	754	326	86	34	1200
A2R5	5	4.6	754	326	86	34	1200
A4R5	5	8.3	754	326	86	34	1200
A5R5	5	10.2	754	326	86	34	1200
A6R5	5	13.8	754	326	86	34	1200
A2R1	1	4.6	754	326	86	34	1200
A4R1	1	8.3	754	326	86	34	1200
A2R0.5	0.5	4.6	754	326	86	34	1200
A5R1	1	10.2	754	326	86	34	1200
A6R1	1	13.8	754	326	86	34	1200
A4R0.5	0.5	8.3	754	326	86	34	1200
A5R0.5	0.5	10.2	754	326	86	34	1200
A6R0.5	0.5	13.8	754	326	86	34	1200
A2R0.1	0.1	4.6	754	326	86	34	1200
A4R0.1	0.1	8.3	754	326	86	34	1200
A5R0.1	0.1	10.2	754	326	86	34	1200
A6R0.1	0.1	13.8	754	326	86	34	1200

**Table 4:** Fault data description 2.

Label	Fault Setting		Sample Size		
	Fault Resistance (Ω)	Shorted Turn Ratio (%)	Training	Testing	Total
A1R5	5	2.78	840	360	1200
A3R5	5	7.41	840	360	1200
A1R1	1	2.78	840	360	1200
A7R5	5	16.67	840	360	1200
A1R0.5	0.5	2.78	840	360	1200
A3R1	1	7.41	840	360	1200
A3R0.5	0.5	7.41	840	360	1200
A7R1	1	16.67	840	360	1200
A1R0.1	0.1	2.78	840	360	1200
A7R0.5	0.5	16.67	840	360	1200
A3R0.1	0.1	7.41	840	360	1200
A7R0.1	0.1	16.67	840	360	1200

**Table 5:** Hyperparameters to be optimized.

Hyperparameters	Search Intervals	Data Types	Transform	Best Result
*L_init_*	[1 × 10^−6^, 9 × 10^−4^]	real	log	4.9947 × 10^−4^
*M*	[0.85, 0.98]	real	log	0.9237
*L*_2*R*_	[1 × 10^−10^, 1 × 10^−2^]	real	log	1.4013 × 10^−10^
*N*	[1, 4]	integer	none	1

**Table 6:** The average accuracy and training time for each algorithm.

Method	Average Time	Test Accuracy (%)		
		Constant	Dynamic	Average
LSTM	7′50″	74.97 ± 10.10	34.58 ± 12.76	66.89 ± 19.69
Bi-LSTM	19′33″	82.50 ± 7.04	61.33 ± 10.24	78.26 ± 11.40
CNN	3′08″	78.39 ± 5.04	37.53 ± 3.06	70.22 ± 17.82
Res	4′22″	92.14 ± 6.87	91.05 ± 2.39	91.92 ± 6.13
TL1	2′15″	96.89 ± 2.49	96.66 ± 0.60	96.85 ± 2.20
TL2	1′42″	96.60 ± 2.62	96.01 ± 1.52	96.49 ± 2.38
TL3	1′37″	95.41 ± 2.91	95.38 ± 0.08	95.41 ± 2.57
TL4	2′24″	96.07 ± 2.78	95.38 ± 0.62	95.94 ± 2.48

**Table 7:** The comparison of the five methods.

Label	Res (%)	TL1 (%)	TL2 (%)	TL3 (%)	TL4 (%)
Time	40′5″	37′46″	28′16″	26′28″	40′12″
ACC	94.94	98.40	97.22	93.76	98.03
HL	92.33	95.70	94.15	84.44	94.75
A1R5	91.44	96.58	94.32	88.89	96.69
A3R5	90.07	98.89	97.23	92.29	98.06
A1R1	91.17	97.91	97.23	93.74	97.78
A7R5	97.41	98.90	98.63	97.41	98.63
A1R0.5	93.17	97.78	95.82	92.43	96.85
A3R1	95.15	98.49	96.32	91.68	98.04
A3R0.5	93.26	98.46	95.51	93.21	98.04
A7R1	99.03	99.58	99.45	98.76	99.58
A1R0.1	94.58	98.74	96.94	93.65	97.46
A7R0.5	98.89	99.17	99.59	97.26	99.17
A3R0.1	98.33	99.72	99.03	96.54	99.58
A7R0.1	99.17	99.16	99.59	97.66	99.72

## Data Availability

The data presented in this study are available on request from the corresponding author. The data are not publicly available due to the follow-up studies are ongoing.
